# Simplified Approach for Preparing Graphene Oxide TEM Grids for Stained and Vitrified Biomolecules

**DOI:** 10.3390/nano11030643

**Published:** 2021-03-05

**Authors:** Anil Kumar, Nayanika Sengupta, Somnath Dutta

**Affiliations:** Molecular Biophysics Unit, Indian Institute of Science, Bangalore 560012, India; anilimac3@gmail.com (A.K.); nayanika.268@gmail.com (N.S.)

**Keywords:** TEM, graphene oxide, Cryo-EM, SEM, AFM, MsDps, apoferritin

## Abstract

In this manuscript, we report the application of graphene oxide (GO) in the preparation of cryo-electron microscopy (cryo-EM) and transmission electron microscopy (TEM) grids. We treated GO with water and organic solvents, such as, methanol, ethanol and isopropanol separately to isolate significantly large GO monolayer flake to fabricate the grids for cryo-EM and TEM study. We implemented a simplified approach to isolate flakes of GO monolayer for constructing the TEM grids, independent of expensive heavy equipment (Langmuir–Blodgett trough, glow-discharge system, carbon-evaporator or plasma-cleaner or peristaltic pumps). We employed confocal microscopy, SEM and TEM to characterize the flake size, stability and transparency of the GO monolayer and atomic force microscopy (AFM) to probe the depth of GO coated grids. Additionally, GO grids are visualized at cryogenic condition for suitability of GO monolayer for cryo-EM study. In addition, GO-Met-H_2_O grids reduce the effect of preferred orientation of biological macromolecules within the amorphous ice. The power-spectrum and contrast-transfer-function unequivocally suggest that GO-Met-H_2_O fabricated holey grids have excellent potential for application in high-resolution structural characterization of biomolecules. Furthermore, only 200 movies and ~8000 70S ribosome particles are selected on GO-coated grids for cryo-EM reconstruction to achieve high-resolution structure.

## 1. Introduction

Graphene is a carbon monolayer [1,2,3,4,5]. The Graphene-based research has also attracted considerable amount of attention in the research community since Novoselov et al. first discovered the preparation and isolation techniques of graphene monolayer in 2004 [6]. Graphene and modified graphene materials have become extremely popular in the research community due to their extraordinary physical, chemical, mechanical and electronic properties [3,4,5]. Different modified graphene derivatives, like functionalized graphene, fluorinated graphene, graphene oxide and reduced graphene oxide have enormous applications in industry [7,8,9]. GO is transparent like graphene, but it is highly hydrophilic and water soluble [10,11]. However, preparation and isolation of GO flake is a challenging task. Hybridization of graphene undergoes a change from sp^2^ to sp^3^ in presence of the highly oxidizing agents KMnO_4_ and H_2_SO_4_/H_3_PO_4_ [12,13,14]. Through this chemical process, the carbon atoms of graphene are modified with oxygen atoms to form different types of functional groups like hydroxyl, ketone, carboxyl and epoxide groups. The basal plane of GO monolayer forms epoxide groups, whereas the edges are fabricated with hydroxyl, ketone and carboxyl groups [15,16]. These additionally fabricated oxygen atoms of GO flake change the charges of the entire GO monolayer and impart hydrophilicity to the single-layered array. However, GO monolayers interact strongly with H atom of -COOH or -OH functional group through stable H-bonding that holds multiple GO layers together. Previous studies showed that GO is decorated with highly electronegative oxygen atoms, which render GO a greatly hydrophilic and water-soluble material [15,16]. This property of GO attracts a significant interest in various research fields, like energy storage and battery industry, hydrogen production, biosensor, biomedical industry, drug-carrier and solar cell industry [17,18,19,20,21]. Additionally, GO is extremely thermo-stable in presence of a strong electron beam [3,4,5]. Therefore, GO monolayer could be used to prepare GO coated TEM grids to avoid thermal drift at cryogenic temperature; although it has remained difficult to isolate large GO flakes due to multiple H-bonding [22] between H_2_O molecules and the different functional groups of GO monolayer. However, there have been some attempts to prepare GO/graphene coated grids for cryo-EM [23,24,25,26,27].

After resolution revolution, cryo-EM has become an extremely eminent structural biology tool for high-resolution structure determination, where biological samples are embedded at different orientation in the thin amorphous ice layer [28,29]. Generally, 2D images of frozen hydrated biological macromolecules are used to perform the high-resolution 3D reconstruction. Among the reported 13,827 cryo-EM maps in EMDB (https://www.ebi.ac.uk/pdbe/emdb/statistics_main.html/), despite of the advancement in microscopic optics, direct-electron detector and automated data acquisition software, only ~49% of structures have been resolved beyond 5 Å due to common barriers such as preferred orientation, adsorption of the sample to air-water interface, particle aggregation, high background from grid coating and poor affinity of the sample for grids. To overcome these potential barriers, amorphous carbon is used most often as a support film [30,31,32], which gives extra stability to the amorphous ice layer. However, amorphous carbon supports pose persistent bottlenecks to high resolution structure determination by producing strong background in the cryo-EM images, wide amount of drift due to strong electron beam exposure and preferred adsorption of samples at the air-water interface [32,33]. Over the previous decade, several methods, like exfoliation, chemical treatment (highly acidic or basic solutions) and organic solvent treatment, have been developed to isolate GO monolayer [10,34,35,36,37,38,39,40,41]. Recently, few researchers deployed functionalized graphene oxide to prepare the grids and cryo-EM sample [4,23,24,42,43,44,45,46], although overall GO isolation procedure is expensive, tedious and time consuming. However, Graphene-based materials or graphene treated with water/methanol mixture have not been rigorously tested for preparing grids for room temperature TEM or cryo-EM study. There are some studies suggesting that GO could be isolated as a very large flake size in presence of water/methanol mixture as compared to water alone [38,47,48,49], although most of studies were only focused on the isolation of the GO flake and size of the flake. The exact size of the GO flake, its stability and the behavior of biological macromolecules on such GO supported grids and application of the resultant GO monolayer to prepare TEM/SEM/Cryo-EM samples have not been studied. In addition, there is a lack of evidence of the behavior of biological samples in the presence of methanol treated GO coated copper grids Therefore, we have employed a simplified and inexpensive technique and performed systematic studies to isolate and characterize GO monolayer with considerably larger flake size to prepare cryo-EM and TEM grids.

In this current study, we clearly establish that our method is able to isolate reasonably large GO flake, which could be successfully used to coat cryo-EM grids and GO-coated Cu grids for high-resolution structural characterization of biological macromolecules. There is no requirement for expensive heavy equipment (e.g., Langmuir–Blodgett, carbon-evaporators, peristaltic pumps, multiple accessories, graphite rods and expansive gases) to prepare flakes. Furthermore, we implemented different imaging techniques, like AFM, SEM, TEM, cryo-TEM and confocal microscopy to illustrate the planarity, stability and transparency of the GO monolayer in presence of organic solvent, which affirms that organic solvent treated GO monolayer is superior in behavior. Furthermore, we have noticed that the adsorption rate of biological macromolecules on methanol treated GO grids is extremely high, making it a specimen enrichment platform which facilitates the visualization and structural characterization of biological samples with very low yield.

## 2. Materials and Methods

### 2.1. Materials

Graphene oxide (Sigma-Aldrich 763705, St. Louis, MI, USA); water was purified with a Milli-Q gradient system (Millipore, Billerica, MA, USA); Quantifoil R2/2 Cu 300 mesh holey carbon grids (Quantifoil^®^ Micro Tools GmbH, Jena, Germany); formvar carbon copper 300 mesh grids (Ted Pella Inc., Redding, CA, USA); Copper Veco grids 300 mesh (Ted Pella Inc., Redding, CA, USA); methanol (AS059 Himedia, Mumbai, India).

### 2.2. UV–VIS Spectroscopy Analysis

UV–VIS spectroscopy analysis was preformed using Eppendorf kinetic bio-spectrophotometer (Eppendorf BioSpectrometer^®^ kinetic 230 V/50-60 Hz, Hamburg, Germany) with wavelength of 220 to 400 nm using quartz cuvette at room temperature. The aqueous suspension of graphene oxide treated with methanol with ratio of 1:9 and graphene oxide with water with the ratio of 1:9 was used as the sample, and the organic solvent was a mixture of water and methanol in 1:5 ratio, and pure Milli Q water was used as the blank. The spectra of these two conditions at 0.02 mg/mL of concentration were recorded at the wavelength of 220 to 400 nm. The aqueous suspensions of graphene oxide treated with organic solvent with ratio of 1:9 and graphene oxide with water with the ratio of 1:9 were sonicated in water bath sonication for 30 min followed by two rounds of centrifugation. The spectra of sonicated samples were also recorded before and after centrifugation.

### 2.3. Confocal Laser Scanning Microscope

Confocal laser Scanning Microscope (Leica TCS SP8 system, Wetzlar, Germany) was performed with different sources of lasers and wavelengths (Diode laser 405 nm, multi Argon laser (458, 488, 514 nm), DPSS 561 nm, HeNe laser 633 nm and with the objectives of 10×/0.40 dry, and 63×/1.4 oil, with optical zoom of 1×. All the images were captured at 1024 × 1024 format with the Z interval of 0.3 micrometer and tile scan were captured at 1432 × 1400 format with Z interval of 3 micrometer. DIC image was captured using Zeiss LSM 800 with the objective of 100×/1.4 oil.

### 2.4. Atomic Force Microscopy (AFM)

All the AFM analysis were performed by using Park NX10 AFM (Park Systems, Suwon, Korea) in Non-contact scanning mode under ambient conditions using ACTA silicon probe with the resonance frequency of 300 kHz and the nominal spring constant 40 N/m. All the AFM imaging is performed at 256 × 256-pixel resolution. All the image analysis, line profiling, and 3D rendering were prepared by Park System XEI version 1.7 AFM image analysis and processing program.

### 2.5. High-Resolution TEM and SEM Analysis of GO-H_2_O and GO-Met-H_2_O

High resolution TEM imaging (FEI, Hillsboro, USA) was performed using 120 kV Tecnai T12 electron microscope equipped with side-mounted Olympus VELITA (2 K × 2 K) CCD camera. GO-H_2_O and GO-Met-H_2_O coated carbon grids were imaged at 120 kV at different magnification range 4200×–63,000×. Similarly GO solution was directly deposited on Cu-grids without any carbon support films for negative staining purposes. Diffraction pattern of GO monolayer and multilayers were recorded at diffraction mode using T12 microscope. Furthermore, the GO-coated Cu-grids were analyzed with JEOL IT-300 (JEOL. Ltd., Tokyo, Japan) scanning electron microscopy. Data were collected at 30 kV high vacuum mode at 10,000× magnification using secondary electron detector (SED).

### 2.6. TEM Grid Preparation with GO-Support Film for Negative Staining and Negative Staining Sample Preparation and Data Collection

300 mesh Cu-grids without any carbon support film was selected to prepare TEM grids with GO-support film. GO-H_2_O and GO-Met-H_2_O solution was directly deposited on TEM grids without any carbon support film, and grids were dried at room temperature overnight. These GO coated grids were directly used for negative staining. Three different biological samples, *E. coli* 70S Ribosome (NEB P0763S), *Vibrio cholerae* Cytolysin and MdtB from *E. coli* were visualized using this GO-coated grids. Standard protocols were followed to prepare negative staining grids, except the glow discharge step before staining. Briefly, 3 µL samples were incubated for 30–60 s on the copper grids coated with methanol-water treated graphene oxide. Excess buffer and bio sample was blotted, and negative staining was executed using 2% uranyl acetate. The entire grids with biological samples were screened at 4200× low magnification to visualize the GO monolayer formation and stability in presence of biological sample and staining solution. Additionally, maximum grids squares of GO-coated grids were inspected at higher magnification (20,000×) to analyze the biological sample distribution on GO support film. The imaging was performed at room temperature using a 120 kV Tecnai T12 electron microscope at 120 kV at pixel size 2.54 Å using side-mounted Olympus VELITA (2K × 2K) CCD camera.

### 2.7. GO-Met-H_2_O Grids Cryo Stability Test

GO-Met-H_2_O coated Quantifoil R2/2 Cu 300 mesh holey carbon grids were blotted for 5.5 s at 100% humidity and quickly plunged into the liquid ethane using FEI Vitrobot IV plunger. The Cryo stability test of GO-Met-H_2_O layer was performed using Thermo Scientific™ Talos Arctica operating at 200 kV on K2 Summit Direct Electron Detector using counting mode. Qualitative assessment was done by focusing the grid holes at 42,200× magnification (1.2 Å/pixel) and exposing with different time intervals (8 s–200 s) at the cumulative dose rate up to 100 e^−^/Å^2^ /s To quantitate the area of damage and vulnerability of the layer, a series of doses were subjected to different areas and the pixel-wise damage was noted. Additionally, several GO-coated holes were exposed with lower electron dose 30 e^−^/Å^2^ for 15–120 s and 80 e^−^/Å2 for 15–120 s to observe the electron beam damage at low electron dose. The series of total electron dose exposure ranged from 3000 e^−^/Å^2^ to 20000 e^−^/Å^2^, and overall damage of the GO layer was measured based on the total amount of electron dose that is required to damage the GO monolayer. However, data was acquired at low magnification (5200× at 9Å/pixel size) after exposing the holes at different electron dose for different time duration. Low magnification image acquisition is required to see the overall damage of the holes after beam damage.

### 2.8. Cryo-EM Sample Preparation and Data Collection

Quantifoil R2/2 Cu 300 mesh holey carbon grids were glow discharged for 90 s at 20 mA, and freshly prepared graphene oxide treated with water-organic solvent was added to glow discharged grid before sample preparation. Freshly prepared *E. coli* 70S ribosome, MsDps2 and apoferritin protein samples (3 μL) were added to the grids and incubated for 10 s before plunging into the liquid ethane. The grids were blotted for 3 s at 100% humidity and quickly plunged into the liquid ethane using FEI Vitrobot IV plunger.

### 2.9. Cryo-EM Data Acquisition

Cryo-EM data acquisition was performed using Thermo Scientific™ Talos Arctica transmission electron microscope at 200 kV equipped with K2 Summit Direct Electron Detector. Images were collected automatically using LatitudeS automatic data collection software (Gatan Inc, Pleasanton, USA) at nominal magnification 42000× at the pixel size 1.2 Å at specimen level. Total electron dose of about 40 e^−^/Å^2^ at the defocus range of −1.25 μm and −3.5 μm at a calibrated dose of about 2 e^−^/Å^2^ per frame. Data were recorded for 8 s and a total of 20 frames. Around 200 micrographs were collected for MsDps2, ribosome and apoferritin for further data processing. The beam-induced motion correction of the individual micrographs was performed using “dosefgpu_driftcorr” using MotionCor2 [50] software (UCSF, San Francisco, CA, USA).

## 3. Data Processing

Data processing was mainly performed using RELION 3 [51]. Initially all the motion-corrected micrographs were manually evaluated using e2display.py and e2projectmanajer.py of EMAN2.1 [52] software package (Baylor College of Medicine, Houston, TX, USA), and the best micrographs were considered for further processing. Initially 10,000 particles were manually picked using RELION 3, and the 2D averages were calculated using RELION 2D classification. Best 2D class averages were selected as template for autopicking, and around 192891 *E. coli* 70S Ribosome particles were automatically selected using the RELION autopicking tool (MRC-LMB, UK). After two rounds of 2D classification, about 54,278 *E. coli* 70S Ribosome particles were selected for 3D classification. Only non-bin data (1.2 Å) were used to calculate the final 3D structure. After 3D classification, 7748 projections of *E. coli* 70S Ribosome were selected and imposed C1 symmetry to calculate the final 3D structure using RELION (EMDB ID: EMD-30399). After final iteration, two unfiltered half-maps were used to calculate the resolution, and the resolution of the 3D models of *E. coli* 70S Ribosome were estimated at 0.143 of Fourier shell correlation (FSC).

## 4. Results

### 4.1. Preparation of GO Monolayer and Characterization of GO Monolayer and Multilayer

**Figure 1:**
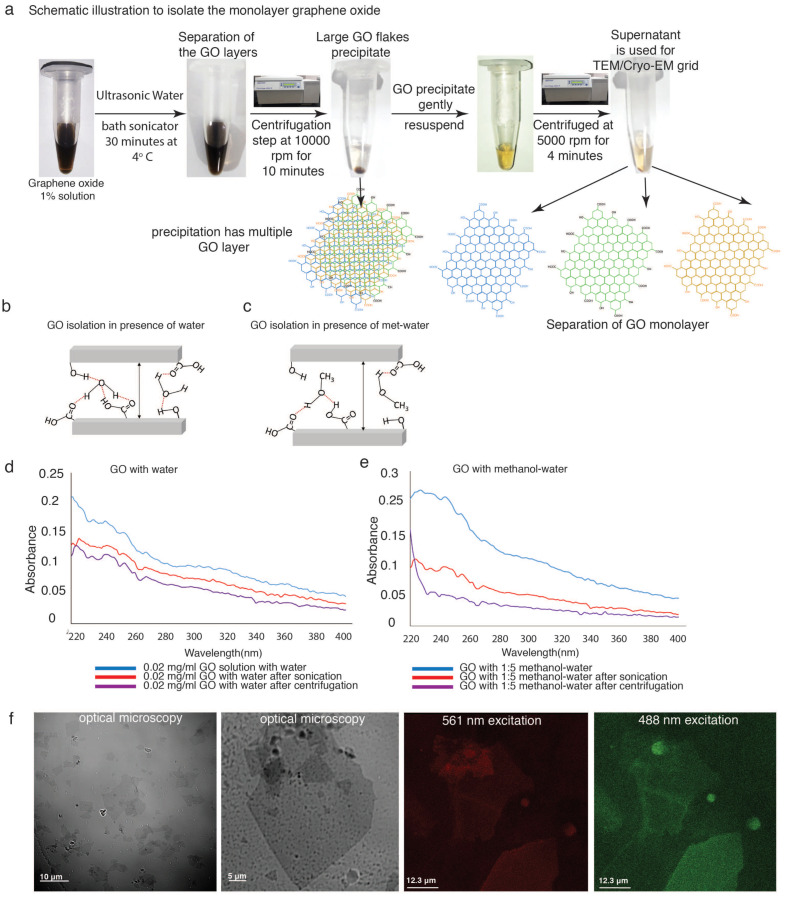
Exfoliation of single layered Graphene Oxide sheets: (**a**) Schematic representation of the workflow for producing Graphene Oxide (GO) monolayer. 1% GO solution is sonicated for 30 min before subjecting it to two rounds of centrifugation. Initial high speed centrifugation at 10,000 rpm to separate and remove the small GO flakes from the solution. The pellet containing multiple layers is gently resuspended and subjected to a 5000 rpm short spin. The supernatant obtained is then used to coat the EM grids. (**b**,**c**) Diagrammatic representation of H-bonding between GO layers and the ambient solvent-water (**b**) and methanol-water (**c**). (**d**,**e**) UV absorption spectra of GO monitored between 220 to 400 nm during the exfoliation process in both the solvent conditions. Red curve shows a distinct fall in A_230_ of GO methanol/water due to segregation of the different populations of GO layers (**e**). (**f**) Suspension of GO in 1:5 methanol-water following centrifugation, is observed with optical and confocal microscopy. GO autofluorescence detected after series of laser excitation at 561 nm, 488 nm, illustrates the presence of large flakes in the size range of 10 µm–70 µm.

The primary objective of this study is to isolate a considerably large GO monolayer using different organic polar solvents to prepare cryo-EM and TEM grids using a simplified procedure without employing any highly expensive equipment. To achieve this target, commercially available graphene oxide solution from Sigma-Aldrich (St. Louis, MI, USA) was used to prepare GO monolayers. Initially 1% GO solution was prepared with water, as well as with 1:5 ratio of methanol/water, ethanol/water and isopropanol/water mixture. Freshly prepared GO solution was sonicated for 30 min in a water bath sonicator (S.V. Scientific) to separate the GO layers. GO solutions were subsequently centrifuged at 10,000 rpm for 10 min, and the supernatant was discarded to remove small (1–5 μm) GO flakes. Furthermore, GO precipitate was gently resuspended using the same solvents as mentioned above and centrifuged at 5000 rpm for 4 min to remove aggregated GO, whereas the supernatant was collected for preparation of TEM grids (Figure 1a). Visual inspection and Ultraviolet-Visible (UV–VIS) spectroscopic analysis of GO dispersed solution in presence of 1:9 water and 1:5 methanol/water (MetOH-H_2_O) was performed (Figure 1d,e). UV–VIS absorption of GO happens within the range of 200–300 nm, whereas maximum absorption is observed at 230 nm due to π electron transition between π–π* orbital of the conjugate hexagonal aromatic rings of GO. UV–VIS spectra indicate that the absorbance of GO-Met-H_2_O is quite low after proper centrifugation, as compared to GO-H_2_O (Figure 1e). Additionally, UV–VIS spectra show that before and after centrifugation, there are no significant changes in absorbance in case of GO-H_2_O, whereas a significant decay of absorbance is observed in case of GO-Met-H_2_O (Figure 1d). Therefore, this spectroscopic measurement allows us to understand the homogeneity of the GO flake, which might be used for TEM grid preparation. However, UV–VIS spectroscopy is unable to determine the size of the GO flake. Therefore, GO supernatant after second centrifugation was visualized by confocal microscopy (Figure 1f) and optical microscopy images show that the supernatant contains GO flakes of size approximately 20–70 µm (Figure 1f).

**Figure 2:**
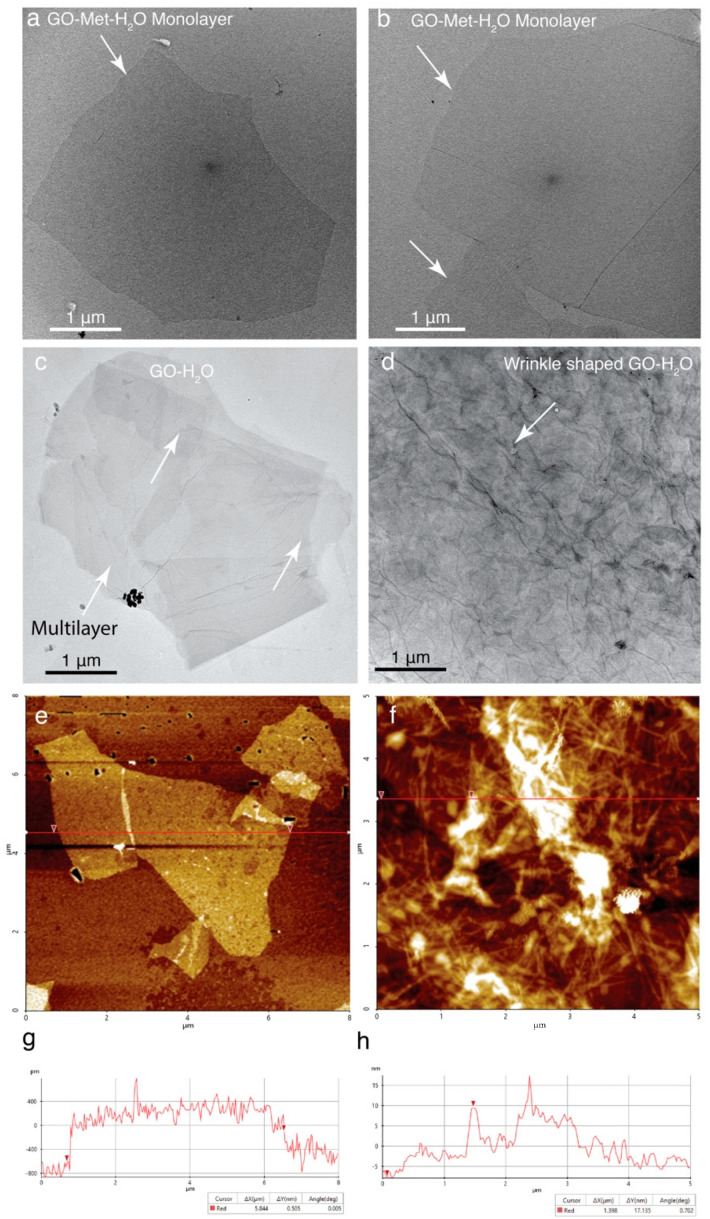
High resolution TEM and AFM studies to elucidate the GO surface properties: (**a**,**b**) High resolution TEM images of GO methanol-water where white arrows point the large monolayers. (**c**,**d**) High resolution TEM images of GO water where white arrows point sporadically distributed and wrinkled multilayers. (**e**,**g**) High resolution AFM image of GO methanol-water monolayer. (**f**,**h**) High resolution AFM image showing the GO water forms multilayer and aggregates.

In order to validate our previous data, high-resolution microscopic studies are required to identify the precise size of the GO flake in presence of H_2_O and Met-H_2_O mixture. Therefore, both the conditions of GO layer were visualized using TEM and AFM. Electron micrographs of two different fields of GO-Met-H_2_O monolayer were imaged (Figure 2a,b). These images distinctly show that methanol treated GO is dispersed as a monolayer with overall size of in the range 20–70 µm. On the contrary, GO flake in presence of H_2_O forms multiple and wrinkled layers (Figure 2c,d). Similarly, AFM data reveal GO-H_2_O show multiple wrinkled layers and folded structure with varied thickness, whereas GO-Met-H_2_O forms a thin single layered large flake (Figure 2e–h).

**Figure 3:**
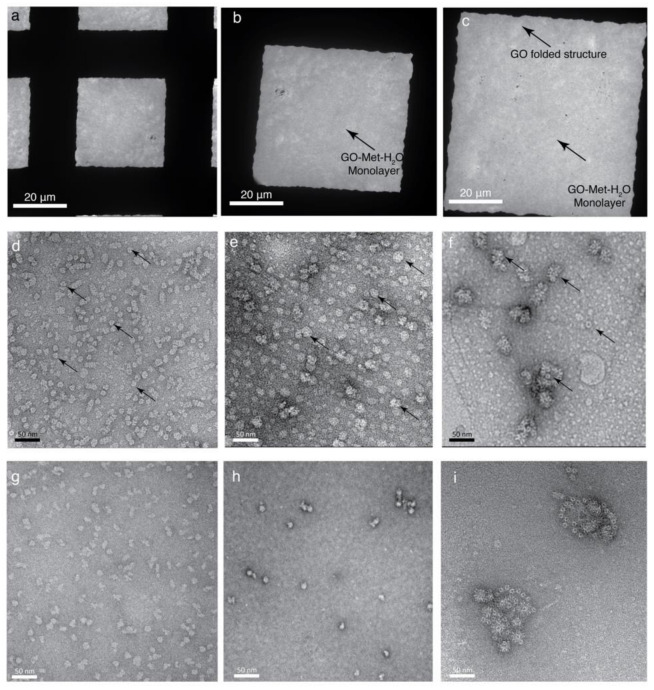
Monolayer GO supported TEM grids for negative staining imaging: (**a**) TEM image shows that the entire grid square is covered with a fine monolayer of GO. (**b**,**c**) Magnified view of each grid square. Arrows point at the near-transparent GO monolayer. A folded structure has been marked to represent complete coverage of the square. (**d**–**f**) Dispersion of various protein samples on GO fabricated TEM grids, observed at 80 K magnification. Arrowheads indicate each protein particle–MdtB (**d**), *E. coli* 70S Ribosome (**e**), VCC (**f**). (**g**–**i**) shows the same sample on carbon coated TEM grids (as control). (**g**) MdtB (**h**) *E. coli* 70S Ribosome and (**i**) VCC on carbon. GO coated TEM grids (**d**–**f**) attracts more biological samples.

Therefore, our current high-resolution TEM studies support our confocal microscopy and AFM studies that GO is dispersed as a large flake (~70 μm) in presence of Methanol-H_2_O mixture. Further, to validate our AFM results, the surface property of GO-coated grids was visualized using SEM and TEM, which illustrates the flattened surface of GO when grids are fabricated with GO-Met-H_2_O (Figure S1a,b,e,f). On the other hand, GO-H_2_O treated grids form an undulating GO surface (Figure S1c,d). The SEM and TEM data show that Met-H_2_O is a suitable solution to isolate large monolayer GO flake, which is able to extend over the entire grid square of holey carbon grids or TEM grids. Therefore, large monolayer flake could be used as a support film for copper TEM grids; usually, TEM grids are coated by lacey carbon support films, carbon films or formvar coated TEM grid. Thus, there is a possibility to use the GO to coat the TEM grids rather than lacey or formvar carbon grids. Hence, we wanted to investigate the GO monolayer interaction and properties with copper TEM grids without any lacey or formvar carbon support film (Figure 3). About 3 µL GO solution was incubated with empty copper grids for 1 min and dried overnight. The freshly prepared grid was visualized by room temperature TEM, optical and confocal microscopy and around 90% of grid squares were found to be covered with GO monolayer (Figure 3a–c and Figure S2). These results suggest that GO monolayer could be used as a support film, and TEM grids could be coated with GO monolayer. This is an extremely interesting finding, which signifies the potential of GO as a support film instead of lacy/formvar/carbon support film for TEM grid—an effective alternative to prepare TEM grids with surface decoration. Since there are no published data reporting the behavior of biological samples in the context of GO-Met-H_2_O, the same was investigated by us. Several biological samples, purified in our laboratory, were imaged in 120 kV TEM at 120 kV using well-optimized staining protocols (Figure 3d–f).

Several biological macromolecules were imaged on the GO support film on TEM Cu-grids, and image quality of same biomolecules was similar to that of the recorded on carbon support TEM grids. Thus, no significant alterations are noticed in biological samples in presence of GO-Met-H_2_O, which suggests GO could be used as a support film to coat TEM grids. Additionally, GO support film grids were not glow discharged or plasma cleaned before adding any biological samples because biological macromolecules easily interact with the hydrophilic GO grids. As opposed to previously available carbon supported grids, which necessitate glow discharge prior to use, an additional hydrophilization step can be omitted for EM grids with this GO layer. Thus, there is no requirement of high-end equipment, like carbon evaporator or glow discharger units to prepare GO coated copper grids. Furthermore, our data show that GO coated TEM grids adsorb more biological samples (Figure 3d–f) than lacy/formvar/carbon support film (Figure 3g–i).

### 4.2. Elucidation of the Surface Property GO-Met-H_2_O and GO-H_2_O Fabricated Holey Grids

**Figure 4:**
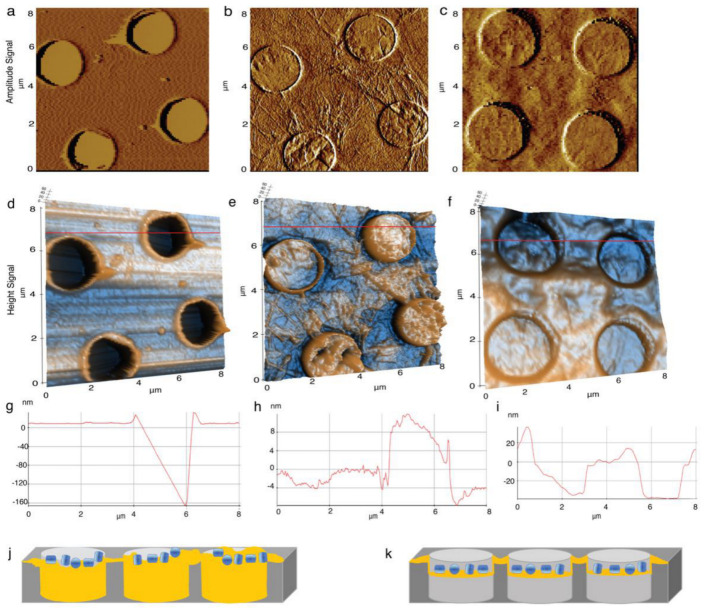
Elucidation of the surface property GO-Met-H_2_O and GO-H_2_O fabricated Cryo-EM grids using AFM (**a**–**c**) 2D amplitude signal of empty holey Carbon grids, GO water flakes and GO methanol-water monolayer supported grids, respectively. (**d**–**f**) 3D height signals recorded from the empty holey Carbon grids, GO water flakes and GO methanol-water monolayer supported grids. (**g**–**i**) Height profile for each grid recorded over a distance of 8 µm shows the available depth of the holes of empty holey Carbon grids (160 nm), GO water flakes (~4–8 nm) and GO methanol-water monolayer (~30–40 nm) supported grids respectively. (**j**,**k**) Schematic diagram shows the coverage inside holes of Quantifoil grids with GO water layer and GO methanol-water surface fabrication, respectively. Schematic diagram shows the GO water multilayer and aggregates almost cover the entire holes (**j**), whereas GO monolayer from methanol-water treatment insert inside the holes to provide uniform depth to carry biological samples and protect the biological samples from air water interface (**k**).

**Figure 5:**
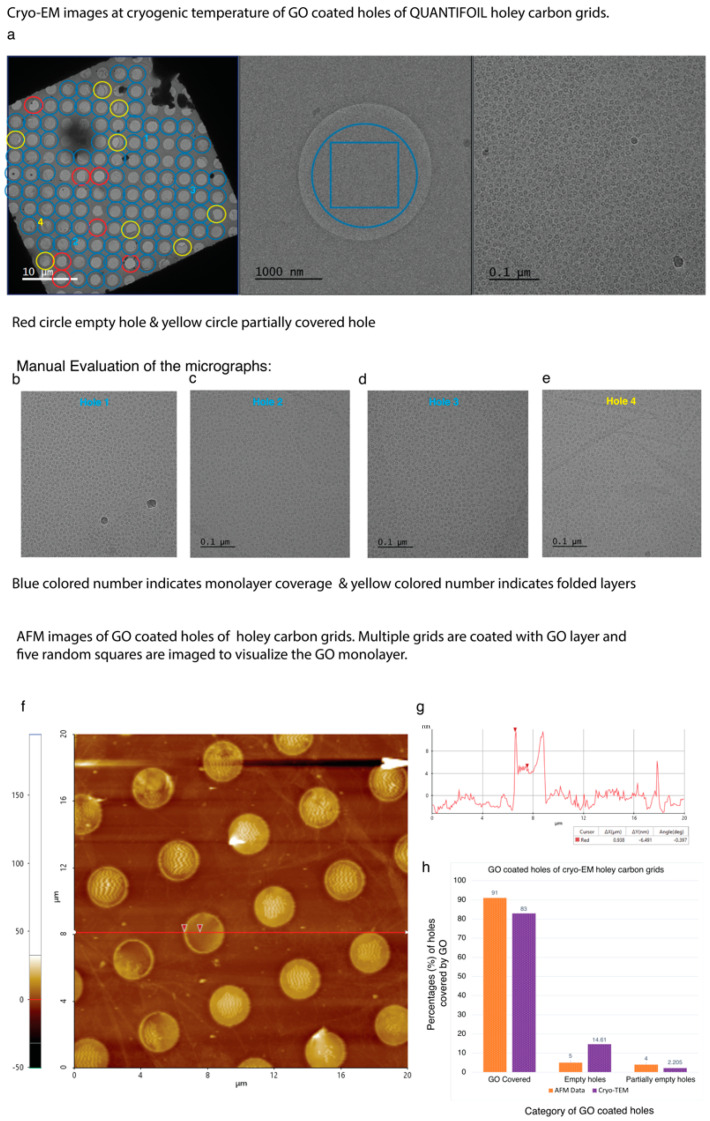
Efficiency of coverage of GO monolayer on holey carbon grids, AFM analysis and SEM, TEM statistical: (**a**) Holey carbon grids are imaged at three different magnifications. At 380× magnification (left panel), each grid square of the holey carbon grid was screened manually and around 83% of holes of each grid square was covered by GO monolayer. Blue circles show GO monolayer, yellow circles are partially covered and red circles are empty holes. At 4300× magnification (middle panel), the coverage of holes from each grid square was manually evaluated at higher magnification. Image is collected at 42,200× magnification. Almost 1500–2000 holes were evaluated manually and almost 83% holes are covered by GO monolayer. (**b**–**e**) Randomly four holes were selected for imaging in image (**a** left panel). Hole1, hole2, hole3 were collected on GO monolayer covered areas (blue circle). Hole4 was collected on partially covered area (yellow circle) (**e**). Hole1, hole2, and hole3 images show that most of the blue holes are covered by GO monolayer. If GO is multilayer is there instead of GO monolayer, multilayer would be visible in high-resolution magnified TEM image, like hole 4 (**e**). Micrographs with folded layer or multilayer are manually screened and removed (**e**). Therefore, the total number of holes covered by GO monolayer are calculated precisely using LatitudeS. Roughly 6 to 14% holes are empty and very few (4–2%) partially empty holes are also observed in this study. (**f**) AFM image of cryo-EM holey carbon grid showing the 91% coverage of GO-methanol-water monolayer. (**g**) Cross section line plot of cryo-EM holey carbon grid showing curve shaped GO decorated holes are extremely suitable to hold biological sample. (**h**) Statistical analysis of GO-methanol-water monolayer coverage over cryo-EM holey carbon grid by AFM analysis, cryo-EM data acquisition software and SEM, TEM.

Atomic force microscopy (AFM, Park Systems, Suwon, South Korea) are the best tools to visualize the surface of the GO-coated grids to elucidate the surface properties and horizontality of GO layers and, therefore, to identify the surface property of GO monolayer within the holes of holey carbon grids. Average thickness of the GO layer of GO-Met-H_2_O is less than 0.5 nm from the AFM line plot (Figure 2e,g), and this thickness is around 5–10 times lower than GO-H_2_O grids (Figure 2f,h). Moreover, planarity of the GO flake is also observed, which shows water treated GO surface is extremely irregular (4–8 nm) (Figure 2f or Figure 4b,e,h) in comparison to methanol-water treated GO layers, where average unevenness of the layer is less than 0.5 nm (Figure 2e,g). We notice an interesting phenomenon of large GO flake: GO monolayer slightly embedded inside the holes of holey carbon grids. On the contrary, multilayer GO-H_2_O also entered and occupied the entire hole of the holey carbon grids (Figure 4b,e). Therefore, wherever GO monolayer is connected with carbon support of holey carbon grids, it forms a flattened GO membrane. However, same GO monolayer does not have any support film when it goes through the 2 µm hole of the holey grid. Therefore, GO monolayer is inserted around 20 nm inside the hole, and less than 0.5 nm thick GO monolayer forms a flat curve shaped architecture inside the hole (Figure 4f,i,k or Figure 5f,g). Under other circumstances, GO-H_2_O produces multilayer flake and aggregates, which also insert within the hole of holey carbon grids, which are unable to generate a GO monolayer (Figure 4b,e,h,j). Therefore, overall thickness of the GO-H_2_O flake implies monolayer and multilayer formation. This current observation suggests that slightly embedded curve shaped GO decorated holes are extremely suitable to hold biological sample, prohibit the absorption of the protein samples completely from grid and protect the protein from air–water interface (Figure 4f,i,k), which is a common problem of GO coated or carbon coated grids [24,32]. On the other hand, holes of holey carbon grids with GO-H_2_O multilayer become uneven, and depth of the hole is reduced due to GO multilayer, which will allow blotting of most of the biological sample randomly from the holes of the grid (Figure 4b,e,h,j). As a result, the amorphous ice formation in GO-H_2_O treated grids is random. This observation suggests that in the presence of GO-H_2_O, most of the holes of holey carbon grids are either dried up or unable to form amorphous ice, or biological samples are mostly blotted out. However, we are unable to visualize the cryo-EM grids at cryogenic temperature using AFM. Therefore, to validate our hypothesis, high-resolution TEM imaging is required at cryogenic conditions. Thus, we have implemented high-resolution TEM imaging, where this phenomenon is observed when different types of GO-coated EM grids are examined with biological sample and data are shown in “Application of GO-coated grids”.

### 4.3. Estimating Stability and Beam Induced Motion of GO-Met-H_2_O Coated CryoEM Grid under Cryogenic Temperature

**Figure 6:**
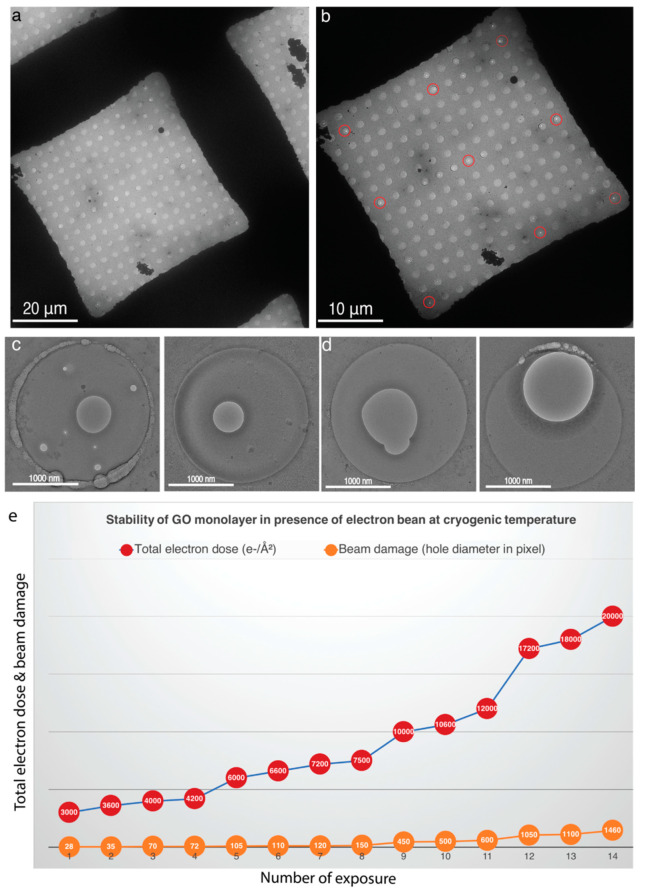
Stability of GO monolayer on electron beam exposure at cryogenic temperature: (**a**) Cryo-TEM image at 115× magnification shows the formation of thin amorphous ice on the GO monolayer supported holey copper carbon grid. Overall amorphous ice distribution is uniform. (**b**) Evenly spread GO monolayer spanning an entire grid square observed at 380× magnification. (**c**,**d**) 4300× magnification of a GO monolayer covered hole after multiple exposures to electron beam for different exposure times from 30–200 s and a dose of 100 e^−^/Å^2^/sec (**c**) and a single prolonged exposure for 200 s and dose of 100 e^−^/Å^2^/sec (**d**) still showing intact GO around exposed areas. (**e**) Graph showing pixel wise damage of GO monolayer with varying electron dose. The red circles indicate the electron dose while the orange circles denote the damage in terms of pixels. It clearly shows that overall damage of GO monolayer is almost negligible even if the total electron dose is 3000–7500 e^−^/Å^2^. Orange curve shows that damages are more severe if GO monolayer is exposed for strong electron dose (10,000–20,000 e^−^/Å^2^).

**Figure 7:**
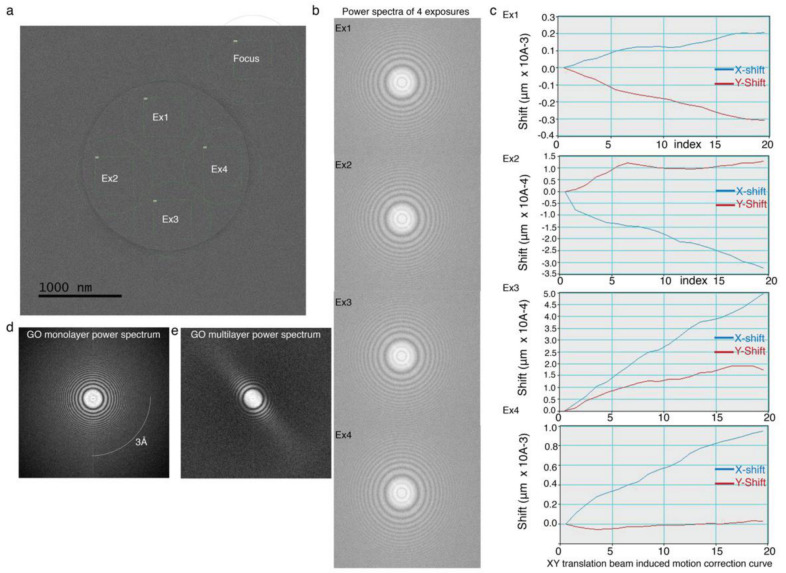
Data acquisition on GO monolayer coated cryo-EM grids: (**a**) Collection of 4 micrographs per hole with a total of 40 e^−^/Å^2^ per exposure. (**b**) Four power spectra from four different exposure and micrographs. All these power spectra are calculated before motion correction. Each of these power spectra is uniform and very stable, and only amorphous ice without any support film, is unable to provide the stable power spectrum for all four images like GO-coated grids. This extra stability is only possible due to stable GO monolayer. (**c**) Thermal drift of four images is corrected using LatitudeS software (GATAN Inc). The XY translation profiles indicate that the beam induced motion was calculated within the multiple frames of acquired movie image and XY translation is less than 5 nm for all four exposures, which clearly indicates the stability of the GO monolayer is extremely high and provides extra stability in presence of electron beam. (**d**) Power spectrum recorded from GO methanol-water coated grid. An arc marked in the figure indicates the 3 Å ring observed in the power spectrum. (**e**) A pronounced loss of signal in one direction is noticed when the power spectrum is recorded from GO water coated EM grid.

Based on our previous results, cryo-EM holey carbon grids were coated with freshly prepared GO solution to visualize the behavior of GO grids at cryogenic temperature and optimized the cryofreezing parameters to prepare the cryo-EM grids of different biological samples. Initially, GO grids without any biological macromolecules were imaged to check the overall coverage, stability, planarity and resolution of GO-Met-H_2_O (Figure 5, Figure 6 and Figure 7) and GO-H_2_O grids. 

High-resolution cryo-EM images of GO-H_2_O coated grids show most of the holes covered by multilayer GO flake and aggregates (Figure S3), which is consistent with our AFM, TEM and SEM results (Figure 4b,e,h,j and Figure S3). These results suggest that H_2_O is unable to disperse large GO flakes. In case of GO-Met-H_2_O after two-step centrifugation, a thin monolayer GO membrane covered 83% of the holes of holey carbon grids manually evaluated using Latitude S automated data acquisition software. (Figure 5a–d). The GO-Met-H_2_O monolayer coverage over holey carbon grid was statistically analyzed by cryo-EM AFM, SEM and TEM (Figure 5a–h and Figure S1a,b,e,f). Cryo-EM analysis and AFM study of GO coated holey grids indicate above 80% holes are covered by GO monolayer, which is good enough for cryo-EM imaging (Figure 5a–g). A statistical analysis was also performed based on the overall coating of GO monolayer on holey carbon grids. Image acquisition was performed at different magnifications to emphasize the size of the GO monolayer and its stability in presence of strong electron beam. Furthermore, beam induced motion has a significant impact in high-resolution cryo-EM imaging, when imaging is performed in amorphous ice [53]. However, beam induced motion is drastically low when image acquisition is performed with carbon coated holey grids [31]. Therefore, different monolayer covered holes were selected to explore the stability of GO monolayer at cryogenic temperature and to identify the beam induced motion of biological sample at the time of cryo-EM imaging. GO coated holes from different areas of holey carbon grid were subjected to multiple exposures to electron beam at different electron doses ranging between 30 e^−^/Å^2^/s to 100 e^−^/Å^2^/s, for 8 to 200 s to illustrate the stability of GO (Figure 6c,d).

In exposures ranging between 8 to 24 s with 30 or 100 e^−^/Å^2^ /s electron dose, no severe damage was observed visually. However, damages were observed in between 30–200 s exposure time at high electron dose. Therefore, one GO covered hole was exposed to electron beam at different positions for 30–200 s exposure time with two different electron dose rate 30 (Placeholder1) or 100 e^−^/Å^2^ /s (Figure 6c,d). Furthermore, one particular area was exposed for longer time, around 200 s (Figure 5d) with higher electron dose rate 100 e^−^/Å^2^/sec. Electron micrograph shows the multiple marks of electron beam interactions with GO membrane (Figure 6b,c). However, even after multiple electron beam exposure, GO membrane does not disrupt completely. Although prolonged subjection to electron beam induces rupture in certain areas of the GO membrane, which is unable to disrupt the entire GO film within that hole (Figure 6d). A more comprehensive test to assess the pixel-wise damage with respect to electron dose shows that a high dose of 3000 e^−^/Å^2^ affects ~28 pixels. This damage becomes pronounced, affecting more than 450 pixels (405 nm), on doses beyond 10,000 e^−^/Å^2^, which are otherwise not applied in biological studies (Figure 6e). Data and curve (Figure 6e) show that extremely high electron dose (3000–4200 e^−^/Å^2^) is required to even form a ~28–72 pixels (33.6–86.4 Å) damage on GO layer. Moreover, graphical representation indicates (Figure 6e) damage is more severe at high total electron dose (100 e^−^ for 200 s) for longer time period, which is completely nonsignificant for biological data acquisition. Generally, total 40–90 e^−^/Å^2^ electron dose is required for 24–6 s for cryo-EM image acquisition, which is an extremely low electron dose for biological imaging. Briefly, high electron dose for short time frame (90 e^−^/Å^2^ for 6 s) or low electron dose for long exposure time (30–40 e^−^/Å^2^ for 10–24 s) is employed for data collection for biological samples. Therefore, an extremely low electron dose is required for bio-imaging, which is unable to disrupt the GO monolayer. Our cryo-stability experiment and curve (Figure 6e) illustrate that GO. Monolayers are super-stable in presence of electron beam for cryo-imaging of biological samples. All these data strongly allude that high energy electron beam could neither disrupt nor dissolve the GO membrane, even after sufficiently long exposures (Figure 6e).

This finding gives us a new opportunity to collect multiple images in a single hole (Figure 7a). Nonetheless, it is important to verify whether the signal-to-noise (S/N) ratio of images is high enough, while multiple images are collected in a single hole. Therefore, four consecutive micrographs were collected from a single hole, and the power spectrum and beam induced motion of the individual frames were calculated to monitor the stability of GO of respective images (Figure 7). The power spectrum and beam induced motion corrections of all four micrographs show that they have similar signal-to-noise ratio (Figure 7b,c). All these data unequivocally support our observation that GO membrane is stable and transparent for imaging of biological macromolecules. The power spectrum serves as a hallmark for identifying single GO layer from multilayer GO (Figure 7d,e) and we noticed that in the latter, the quality of the power spectrum is severely affected due to multilayer in case of GO-H_2_O coated grids (Figure 7e).

### 4.4. Application of GO Monolayer for Cryo-EM Imaging

**Figure 8:**
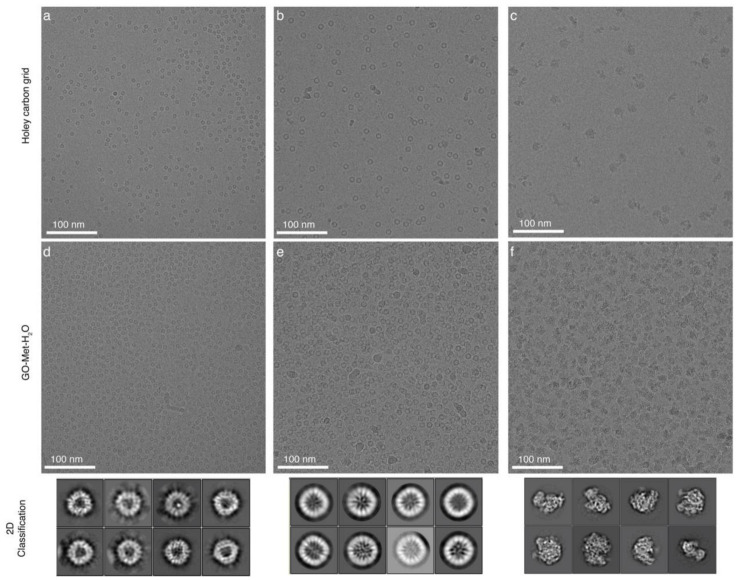
Application of GO methanol-water grid for cryo-EM of biological specimens: (**a**–**c**) MsDps, Apoferritin and *E. coli* 70S Ribosome on conventional holey carbon grid. (**d**–**f**) MsDps, Apoferritin, and *E. coli* 70S Ribosome on GO-Met-H_2_O grid. Relatively planar and flattened GO-Met-H_2_O coated holey grid is able to adsorb more biological samples than conventional holey carbon grid. Lower panel represents the corresponding reference-free 2D class averages of each protein specimen vitrified on our modified GO grid. 0.1 mg/mL of each sample was applied on the grids.

Both types of grids, GO-Met-H_2_O and GO-H_2_O, were used to prepare cryo-EM samples. Three well optimized biological samples, like MsDps (*Mycobacterium smegmatis* DNA-binding Protein from Starved cells), Apoferritin and *E. coli* 70S Ribosome were selected to visualize using cryo-EM (Figure 8). Sample concentration, total amount of samples and other freezing parameters were the same for both the grids. Particles were well distributed, and signal-to-noise ratio of micrographs was very high in both grid types. Surprisingly, we noticed that GO-Met-H_2_O grids adsorb almost 10 times higher biological samples than holey carbon grids (Figure 8a–f). These interesting findings encouraged us to use the GO-Met-H_2_O grids in cases where protein concentration was extremely low. Some biological samples tend to aggregate at higher concentration, and it becomes exceedingly difficult to perform cryo-EM based structural characterization of these kinds of samples. As a user-friendly alternative to this problem, GO-Met-H_2_O adsorbs more biological samples and works efficiently at extremely low protein concentration as compared to conventional grid preparation (Figure 3g–i).

Therefore, GO-Met-H_2_O coated grids would be a better choice for cryo-EM studies of aggregation prone biological samples. Therefore, one such kind of protein samples, EccA1, cytosolic component of bacterial type VII secretion system from *Mycobacterium tuberculosis* was investigated using GO-Met-H_2_O coated grids (Figure S4c). Additionally, GO-Met-H_2_O supported grids facilitate us to resolve the preferred orientation problem of thermostable direct hemolysin, a 72-kDa tetrameric protein (Figure S4a,b). Furthermore, GO-Met-H_2_O coated grids were used to record the images of MsDps, Apoferritin, and *E. coli* 70S Ribosome bio samples for further data processing. Reference free 2D classification of MsDps, Apoferritin and *E. coli* 70S Ribosome shows fairly detailed information and internal structural attributes of these protein molecules (Figure 8d–f). Moreover, a 3D reconstruction of *E. coli* 70S Ribosome was determined to visualize the structural integrity of biological sample on GO-Met-H_2_O grids. 3D reconstruction of *E. coli* 70S Ribosome was successfully calculated at 4.6 Å resolution using extremely low numbers of ~7800 particles and 300 images (Figure S5). Extra stability of GO supported grids provides us this opportunity to resolve a near-atomic resolution structure of bio sample using such low number of particles and micrographs. These data suggest that GO coated grids are well suited for high-resolution structural characterization of biological samples, and methanol does not have any adverse impact on structural or conformational changes of the protein samples. Therefore, our new strategy to prepare cryo-EM or TEM grids with GO support film is an easy and low-cost technique with lucrative commercial application in the future.

## 5. Discussion and Conclusions

In this manuscript, we explored high-resolution TEM, cryo-TEM, SEM and AFM techniques to identify large uniform GO monolayer flake with water and methanol-water mixture. We also report here that there are no significant changes in properties, like transparency, planarity and stability of GO monolayer in the presence of methanol-water mixture. We successfully presented that GO treated with methanol-water mixture is capable to generate larger flake size to fabricate the TEM grids without forming aggregates like water treated GO solution [24]. Additionally, we showed that GO-Met-H_2_O solution could be used to prepare TEM grids with GO support film, which is extremely easy to construct and any heavy expensive equipment like, Langmuir–Blodgett trough; carbon-evaporator or peristaltic pumps, carbon evaporator and plasma cleaner, is non-compulsory. However, Met-H_2_O treatment is extremely essential to disperse the large GO flake, which is quite difficult with only water. This phenomenon is supported by several published data [38,47,48,49]. One explanation for large GO flake formation with methanol as opposed to water is that the latter forms multiple H-bonds between the two H-atoms of H_2_O and the carboxyl, hydroxyl and epoxy functional groups of GO (Figure 1c). Therefore, it is an extremely challenging task to separate two layers of GO, and more energy is required to disperse monolayers. Therefore, GO in presence of water is prone to dissociating into small fragments, and multiple GO fragments interact with each other to produce a large multilayer flake. In the case of methanol-H_2_O mixture, methanol and H_2_O both interact with GO layers. In the presence of CH_3_OH, one H-atom is replaced by an aliphatic -CH_3_ group, which is unable to form H-bonding with any functional groups of GO. Therefore, a lower number of H-bonding in the presence of methanol-H_2_O aids in isolating GO monolayer. Further, distance between two GO layers is higher due to a bulky -CH_3_ group that might in turn ease the monolayer separation procedure (Figure 1c). Intuitively, the presence of bulkier aliphatic groups in ethanol(-CH_2_CH_3_) and isopropanol(-CH(CH_3_)) urges one to explore their potential in dispersing large sheets of monolayered GO. However, our TEM analysis showed that GO flakes with ethanol or isopropanol are folded and wrinkled (Figure S6). This suggests that -CH_2_CH_3_ and -CH(CH_3_) groups disperse extremely large GO flakes, which might be difficult to be maintained as a 2D planar structure. These colossal GO flakes further adopt a folded conformation to maintain the stability of single layer and undergo a wrinkled structure formation. Therefore, after comprehensive screening of organic solvents, we conclude that only methyl(-CH_3_) group is suitable to disperse the GO monolayer fit for TEM and cryo-TEM grids. However, few published manuscripts also focused on the GO and functionalized-GO monolayer separation using only H_2_O [54,55] and various ratios of H_2_O-MetOH mixture [24]. However, Kim et al. were able to isolate the GO monolayer >5 μm; similarly, Palovcak et al. demonstrated the separation of GO monolayer and elucidated a simplified apparatus to coat the cryo-EM grids, which successfully covers 80% percent of the holes, although the target of this study is the development and application of this simplified apparatus to prepare the cryo-EM grids. Nevertheless, all these studies suggest that water-organic solvent (mainly H_2_O-Methanol) mixture is a suitable solution to isolate the GO monolayer. However, none of these studies are approached like this current study, where H_2_O-Methanol mixture is used to isolate the GO monolayer to prepare cryo-EM grids and TEM grids. Furthermore, in this current method, heavy or light equipment are not required to isolate the GO monolayer. Basically, it is a cleaning technique with water-methanol mixture and centrifugation of the solution for two-three times to isolate large GO flakes, where the majority of GO flakes are 20–70 μm. The previous study by Zheng et al. showed that ~20–50 μm GO monolayer functionalized-GO monolayer separations [45,46] could be isolated using low pressure Chemical Vapor Deposition (LPCVD) system successfully. Additionally, NTA modified graphene oxide monolayer was successfully isolated for TEM grids preparation [44], and the overall size of the GO monolayer is between ~16 and 37 μm. However, this current study also indicates that H_2_O-Methanol is suitable to isolate GO flake of almost the same size but using a more simplified method. Our visual inspection, manual screening of individual holes of entire grids (5 grids are screened manually) and statistical analysis suggest that GO monolayer isolated by H_2_O-Methanol is appropriate to coat individual square (50 μm × 50 μm) of holey carbon grids (Figure 5). However, all the GO coated grids were used to determine the cryo-EM structure of proteasome [24,45], GroEL [44], and apoferritin [47]. Nevertheless, none of them were able to characterize a 4.6Å map using only ~7800 single particle projections using GO coated holey grids. Additionally, we have demonstrated that our method is capable to isolate GO monolayer to cover the uncoated Cu-grids for room temperature TEM study. Furthermore, our AFM studies show that GO monolayer is embedded 30–40 nm inside the holes of holey grids, which is able to adsorb significant number of biological samples than GO-H_2_O grids.

Furthermore, our TEM, SEM and cryo-EM studies firmly illustrate that Met-H_2_O treated GO produces a reasonably large and stable GO flake, which is efficient to cover one entire grid square, that is otherwise impossible to achieve with water treated GO solution. Our AFM studies indicate that the large GO monolayer inserts within the holes (Figure 4k) and provides enough depth for proper adsorption of biological samples and protects the samples from air-water interface; thus, there is no requirement of adding sample from the backside of the grid as Palovcak et al. demonstrated [24]. On the contrary, GO-H_2_O produces multilayer GO flakes, which go inside the hole and reduce its depth. This in turn hinders the adsorption capacity of the biological samples. As a result of this, cryo-EM micrographs of biological macromolecules clearly suggest that GO-Met-H_2_O grids adsorb 10 times more biological macromolecules than conventional holey carbon grid in amorphous ice. In several cases, especially for membrane proteins, it is expensive to isolate huge amounts of purified membrane protein sample or complexes. Therefore, this new methodology will help the entire cryo-EM field to prepare GO-Met-H_2_O grids, where protein requirement is 5–10 times lower than conventional cryo-EM sample freezing. Moreover, from the power spectrum, we can see that with GO-methanol-water grids, resolution as high as 3 Å can be reached. Reference free 2D classification of all three biological samples shows that methanol treated GO does not inhibit the high-resolution features, and there are no significant conformational changes or deformities in biological macromolecules due to methanol. In effect, our studies show that GO-Met-H_2_O is highly hydrophilic, more stable in presence of electron beam and a favorable platform for biological macromolecules, in contrast to GO-H_2_O. Furthermore, our findings demonstrate that the GO flake from GO-Met-H_2_O is capable to cover empty grids, and biological macromolecules could be imaged on these grids using heavy-metal staining solution. Moreover, there is no significant conformational or structural changes. Therefore, GO-Met-H_2_O solution could be used to prepare GO supported TEM grids. There are other chemical compounds like NaOH, DMSO, cyclopentanone, acetone, N-Ethylpentedrone (NEP) and N-Methyl-2-Pyrrolidone (NMP) which could be more potent to isolate larger GO flakes than alcohol solvent [56,57,58]. Additionally, several other methods report, GO could be reduced to reduced GO (rGO) and isolate as a monolayer by ascorbic acid (AA), where overall flake sizes are 10–60 μm [59]. GO monolayer could be isolated using a mixture of poly-vinyl alcohol and H_2_O mixture where flake sizes differ from 1–80 μm, although the size of the flakes would be controlled by duration of sonication as well as temperature at the time of sonication [60]. Further investigation is required to explore the ability of these solvents in isolating large planar GO flakes, and it is our next target in the future.

To summarize, we are able to isolate 70 µm large planar monolayer GO flake with Met-H_2_O mixture, which could span the TEM grids and these grids could be used to visualize stained and vitrified biological macromolecules. Our method to isolate GO monolayer is more consistent and easy to use in comparison to GO-H_2_O. Our modified grids can enrich difficult targets for structural analyses, which are otherwise limited by poor expression levels in heterologous systems or concentration dependent aggregation. Therefore, these simplified approaches to prepare EM grids will open up a new avenue for negative staining and cryo-EM grid preparation techniques.

## Data Availability

Cryo-EM 3D reconstruction map of *E. coli* 70S ribosome has been deposited to EM Data Bank (EMD-30399).

## Supplementary Materials

The following are available online at https://www.mdpi.com/2079-4991/11/3/643/s1, Figure S1: Scanning Electron Microscopy to visualise GO methanol-water and GO water layer. Figure S2: Isolation of large monolayer GO flake to cover Negative Staining TEM grid. Figure S3: Water treated GO is distributed as discontinuous, small, monolayered flakes and multi-layered and aggregates. Figure S4: Cryo-EM imaging of different biological samples on GO monolayer. Figure S5: Cryo-EM structure determination of *E. coli* 70S Ribosome vitrified on GO monolayer coated grid. Figure S6: Transmission Electron Microscopic analysis of GO isopropanol-water and GO ethanol-water layer.
