# Supplementary material for: Simplified Approach for Preparing Graphene Oxide TEM Grids for Stained and Vitrified Biomolecules
Source: Nanomaterials (Basel). 2021 Mar 5;11(3):643. doi: 10.3390/nano11030643 (PMC7999706; doi:10.3390/nano11030643)
Supplement: Supplementary file 1 [file nanomaterials-11-00643-s001.pdf]

nanomaterials

Supplementary Materials

Simplified Approach for Preparing Graphene Oxide TEM Grids for Stained and Vitrified Biomolecules

Anil Kumar, Nayanika Sengupta, Somnath Dutta*

Molecular Biophysics Unit, Indian Institute of Science, Bangalore 560012, India; anilimac3@gmail.com (A.K.); nayanika.268@gmail.com (N.S.)

* Correspondence: somnath@iisc.ac.in; Tel.: 080-22933453; Fax: 080-2360 0535

Table of Contents

Figure S1. Scanning Electron Microscopy to visualise GO methanol-water and GO water layer

Figure S2. Isolation of large monolayer GO flake to cover Negative Staining TEM grid.

Figure S3. Water treated GO is distributed as discontinuous, small, monolayered flakes and multi-layered and aggregates.

Figure S4. Cryo-EM imaging of different biological samples on GO monolayer

Figure S5. Cryo-EM structure determination of *E. coli* 70S Ribosome vitrified on GO monolayer coated grid

Figure S6. Transmission Electron Microscopic analysis of GO isopropanol-water and GO ethanol-water layer

Figure S1. Scanning Electron Microscopy to visualise GO methanol-water and GO water layer:

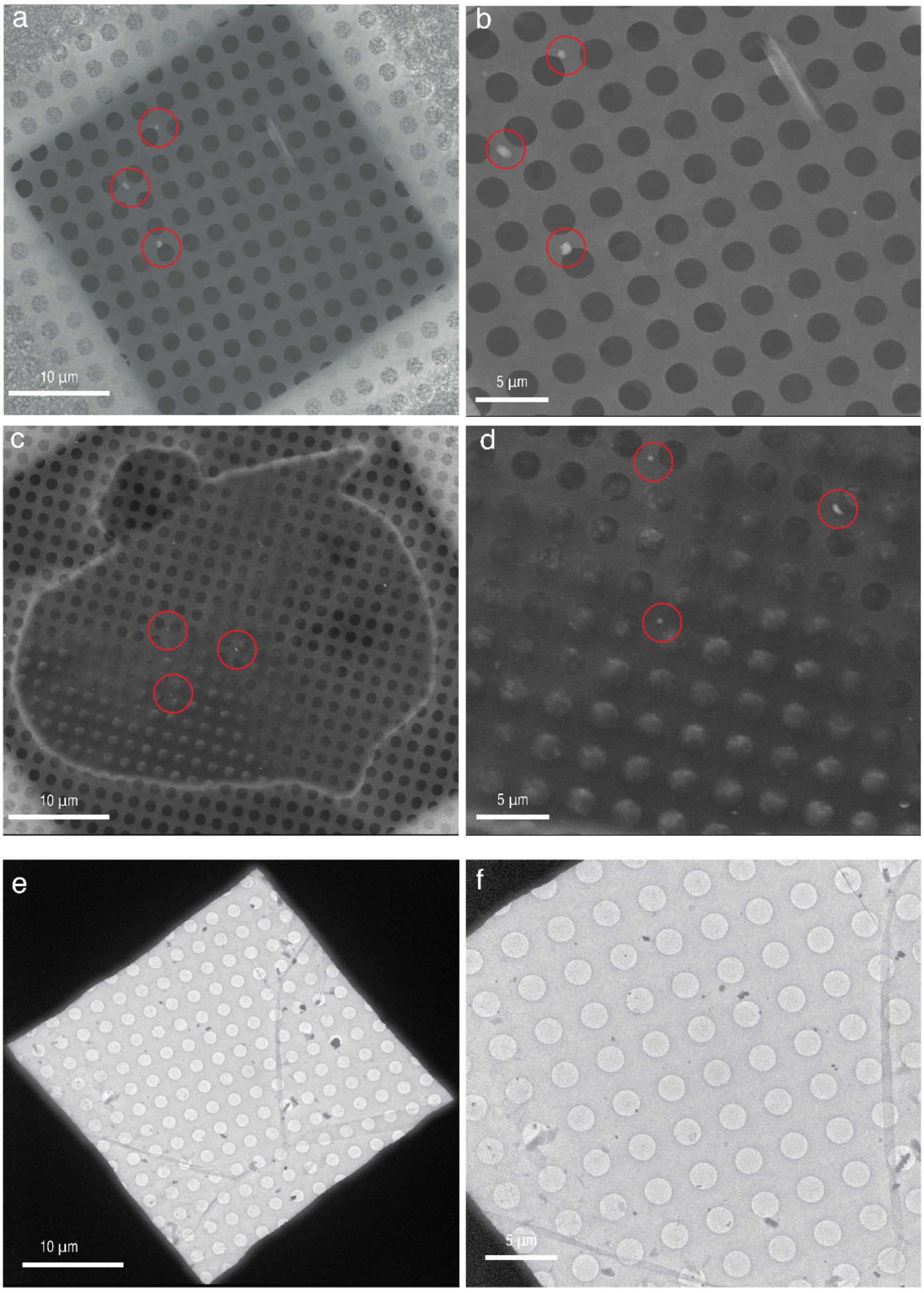

Figure S1. (**a**) Flat and even distribution of GO methanol-water monolayer on a Quantifoil cryo-TEM grid. A magnified image of the same is represented in (**b**). (**c**) shows the coverage obtained on GO water coated Quantifoil grid. A magnified image of the same is represented by (**d**). Red circles highlight images are acquired at same positions for both the grids at different magnification. (**e**,**f**) TEM image GO-Methonal coated holey carbon grid which show 83% of holes are covered.

Figure S2. Isolation of large monolayer GO flake to cover Negative Staining TEM grid:

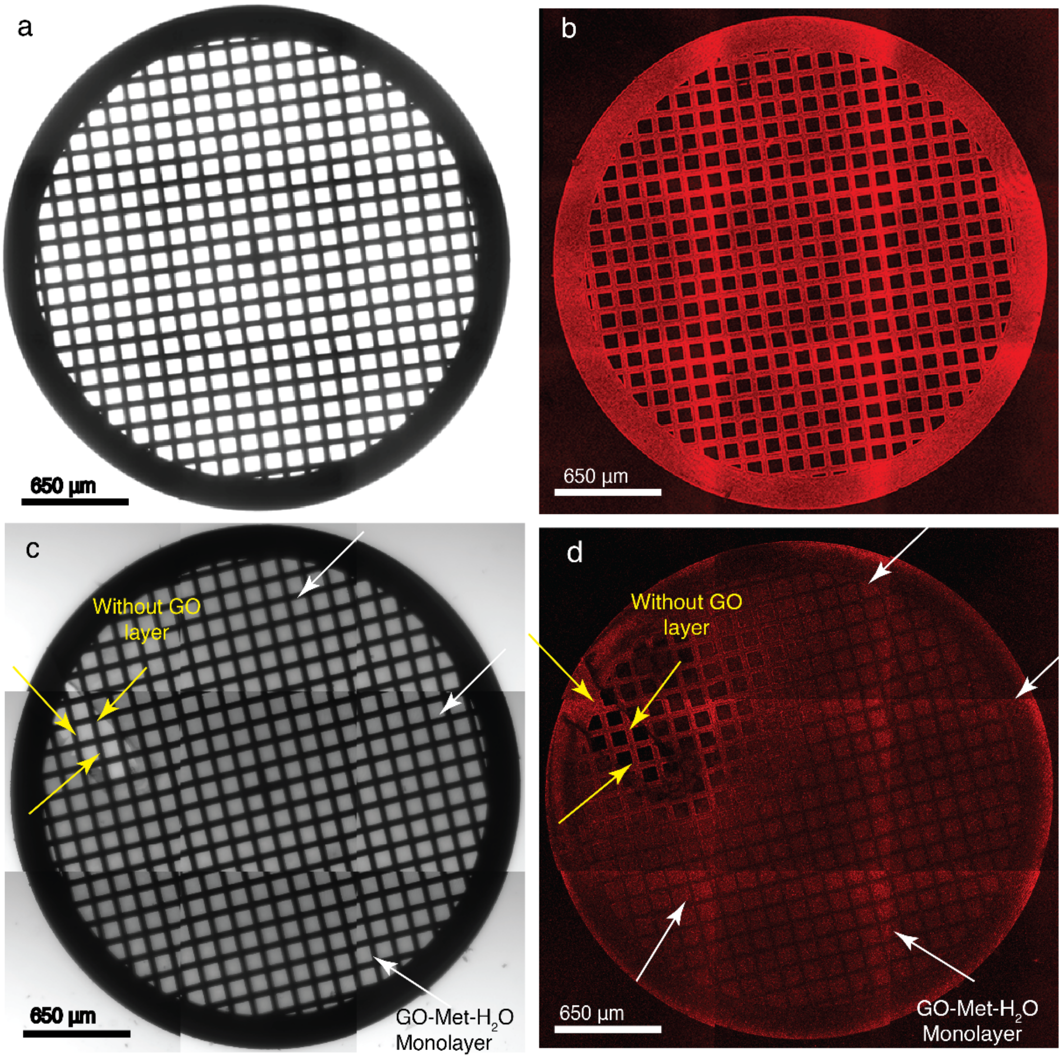

Figure S2. (**a**) Brightfield microscopy image of unsupported Copper TEM grid. (**b**) Confocal Microscopy image of unsupported Copper TEM grid excited at 561 nm. (**c**) Brightfield microscopy image showing almost 90% efficiency of GO flake in covering the entire TEM grid. (**d**) Corresponding confocal microscopy image of supported Copper TEM grid excited at 561 nm. Yellow arrows trace the area of broken layer while continuously distributed monolayer is marked by the white arrows.

Figure S3. Water treated GO is distributed as discontinuous, small, monolayered flakes and multi-layered and aggregates:

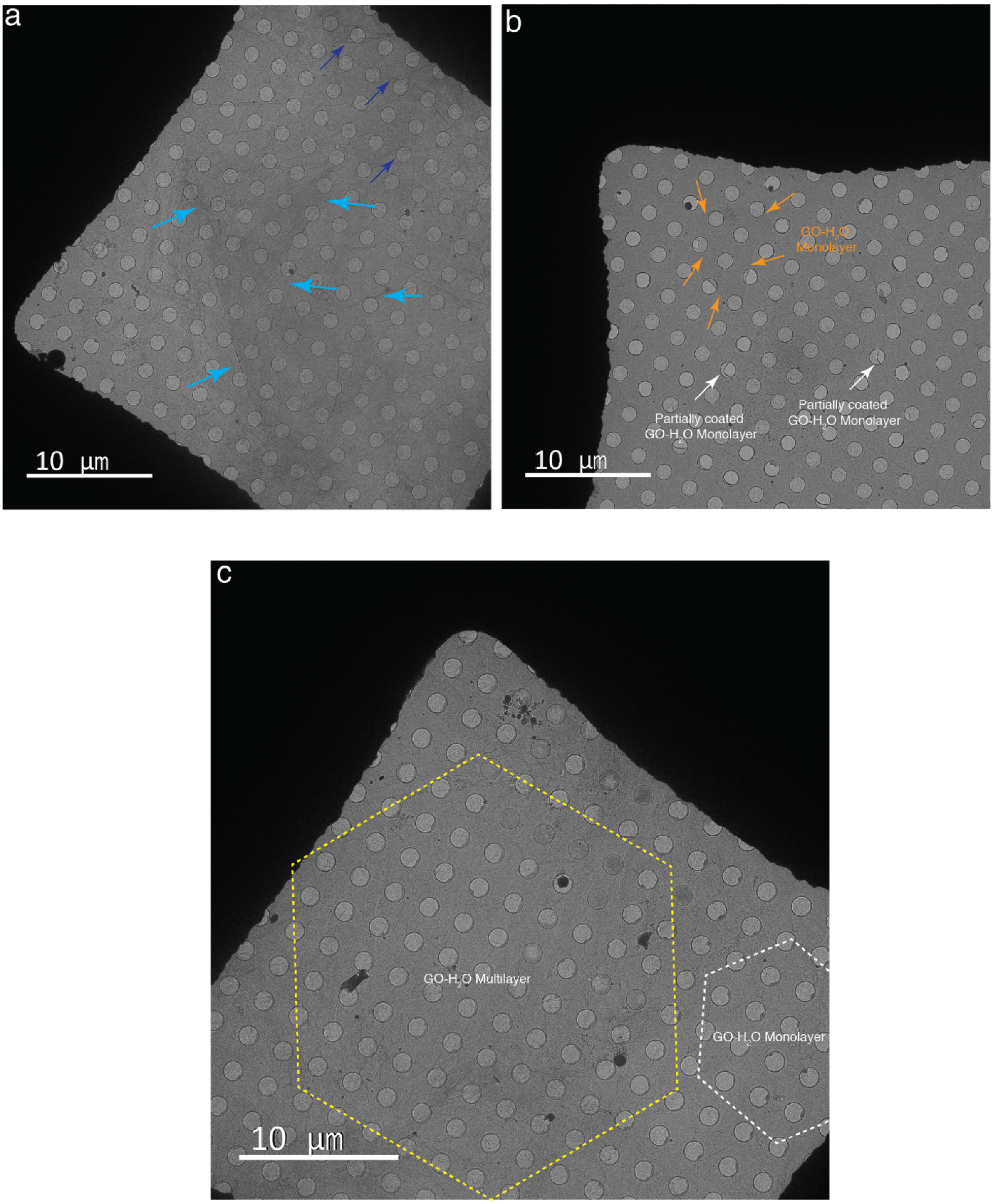

Figure S3. (**a**) Grid square covered with small, multiple sheets of folded GO (light blue arrows) and few monolayers overlapping with each other (dark blue arrows). (**b**) A different grid square shows partially covered holes (white arrows) and a single monolayer spanning less than 10 µm (orange arrows). (**c**) GO in water is predominantly dispersed as multilayers and aggregates (yellow dashed hexagon). A small nearby GO monolayer is visible within the dashed white hexagonal boundary.

Figure S4. Cryo-EM imaging of different biological samples on GO monolayer:

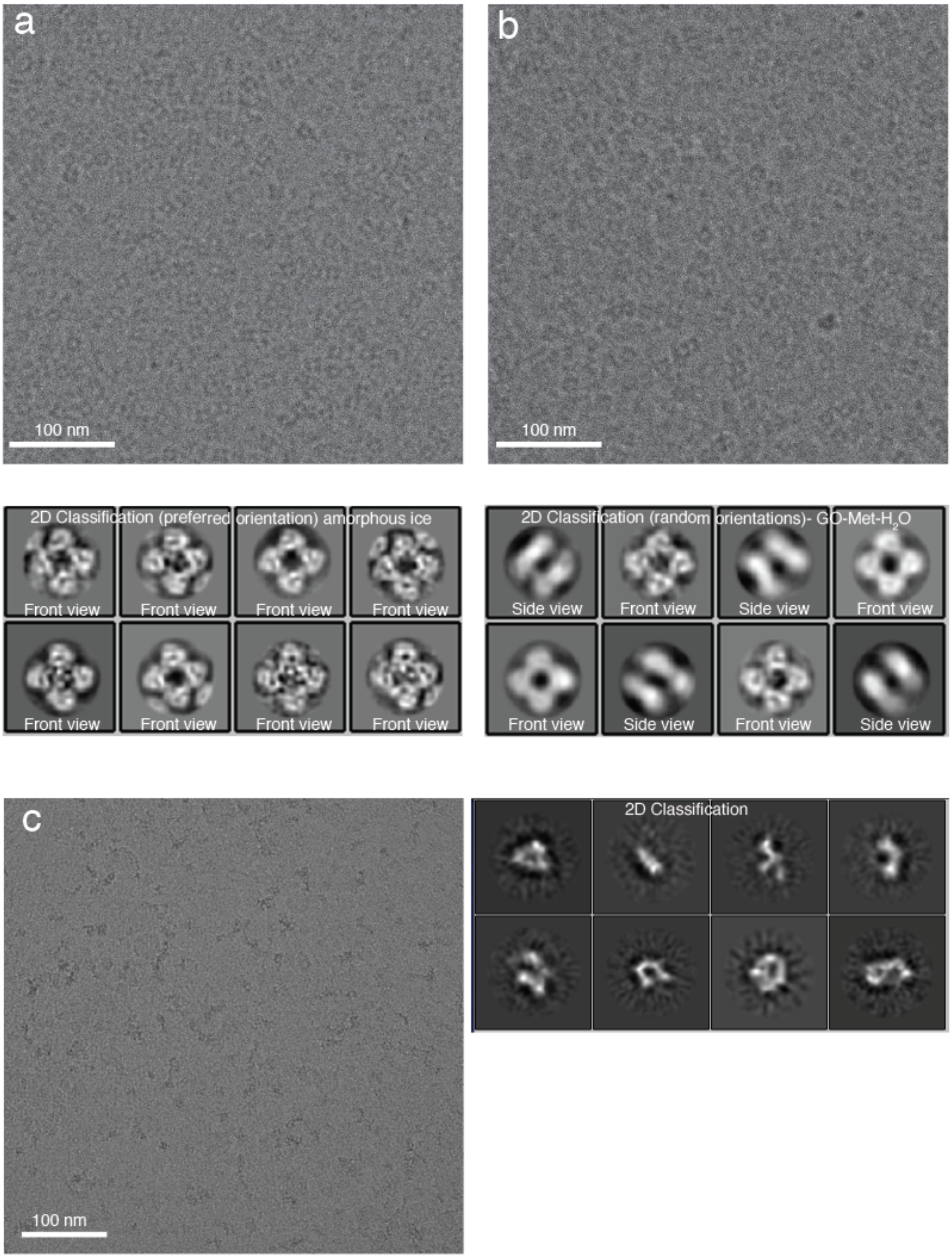

Figure S4. (**a**) Representative micrograph where the preferred views of TDH are visible. Corresponding reference-free 2D class averages comprise top and bottom views of the toxin. (**b**) Various orientations of TDH obtained on GO methanol-water grids. Corresponding reference-free 2D class averages show well represented side views. (**c**) Efficient adsorption of low concentration biological sample (0.05 mg/mL) EccA1 vitrified on GO methanol-water grid – denoted by the micrograph and reference-free 2D class averaging.

Figure S5. Cryo-EM structure determination of *E. coli* 70S Ribosome vitrified on GO monolayer coated grid:

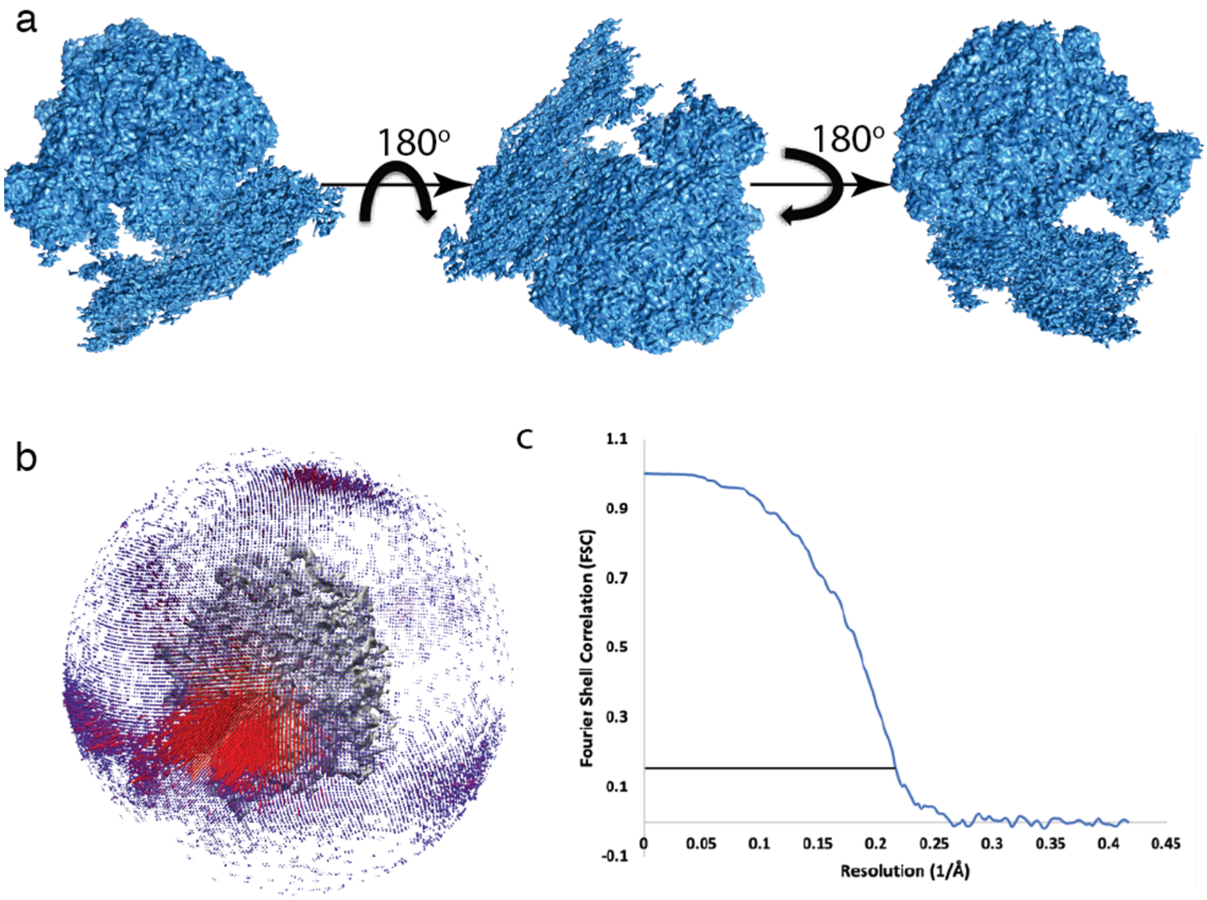

Figure S5. (**a**) 4.6 Å 3D reconstruction of *E. coli* 70S Ribosome. (**b**) Orientation distribution of 70S Ribosome on GO methanol-water grid. Colour contrast signifies the least (blue) and most (red) represented orientations. (**c**) Fourier Shell Correlation curve showing the 4.6 Å resolution, estimated at the cut off value of 0.143 FSC.

Figure S6. Transmission Electron Microscopic analysis of GO isopropanol-water and GO ethanol-water layer:

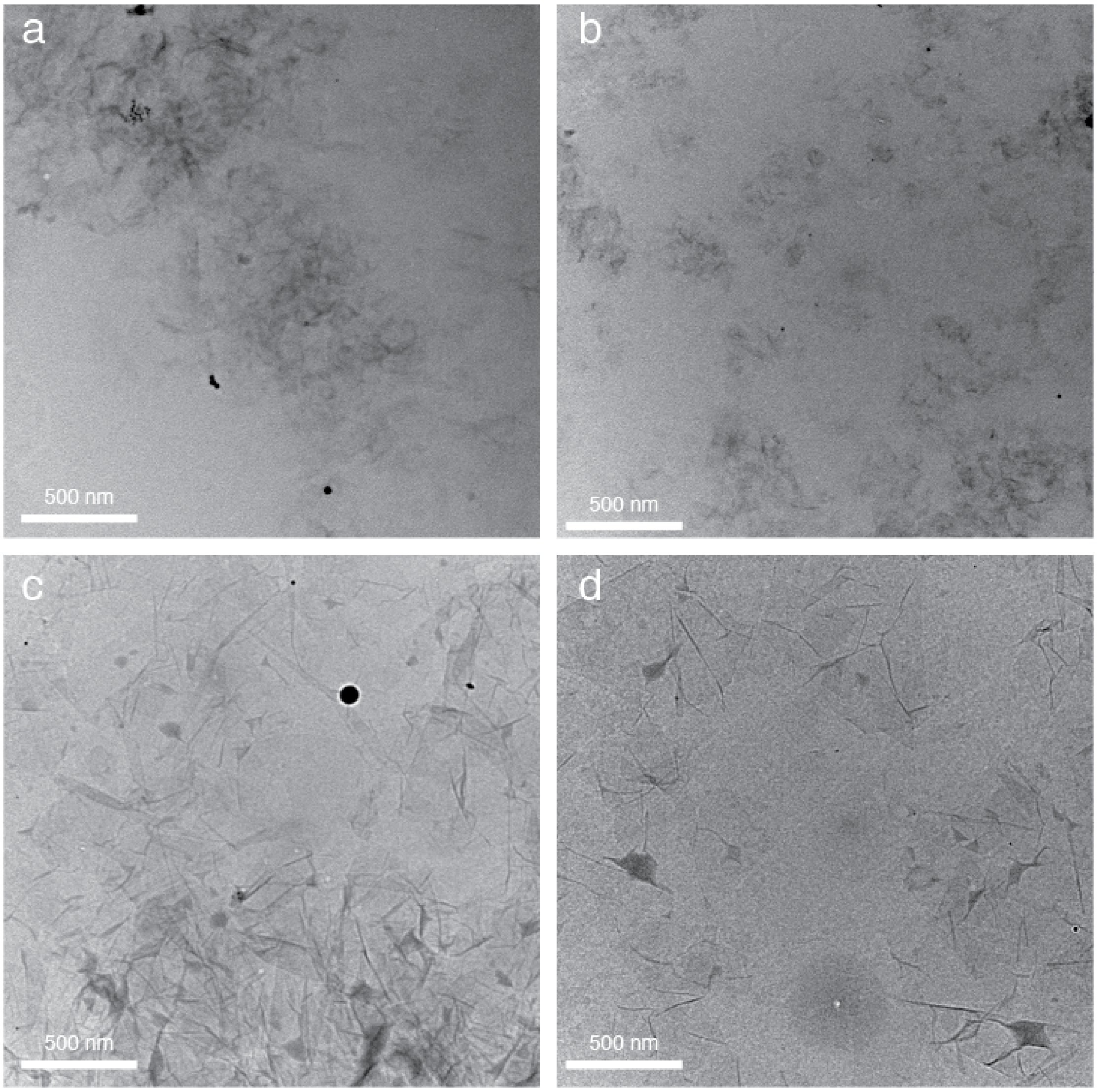

Figure S6. (**a**) (**b**) Two fields showing inhomogeneity in layer formation when GO is dispersed in isopropanol-water. (**c**) (**d**) Wrinkle shaped layers formed by GO ethanol-water.

© 2021 by the authors. Licensee MDPI, Basel, Switzerland. This article is an open access article distributed under the terms and conditions of the Creative Commons Attribution (CC BY) license (http://creativecommons.org/licenses/by/4.0/).
